# Development of a Simplified Protocol for Respiratory Muscle Segmentation in Unenhanced Chest CT and Identification of New Radiomic Biomarkers of Sarcopenia in Lung Diseases: A Retrospective Study

**DOI:** 10.3390/jcm14248712

**Published:** 2025-12-09

**Authors:** Riccardo Picasso, Maria Elena Susi, Giovanni Marcenaro, Marta Macciò, Federico Zaottini, Federico Pistoia, Ludovico La Grutta, Giulia Sollami, Arianna Maggio, Diego Bagnasco, Fulvio Braido, Melissa Ferraris, Ludovica Napoli, Benedetta Bondi, Giulia Carpani, Gaia Vettori, Maurizio Mongelli, Alessia Paglialonga, Carlo Martinoli

**Affiliations:** 1 IRCCS Ospedale Policlinico San Martino, 16132 Genoa, Italy; riccardo.picasso@gmail.com (R.P.); federico.zaottini@hsanmartino.it (F.Z.); federicopistoia1@gmail.com (F.P.); carlo.martinoli@unige.it (C.M.); bennina.bondi@gmail.com (B.B.); fulvio.braido@unige.it (F.B.);; 2 Department of Health Sciences (DISSAL), University of Genoa, 16126 Genoa, Italy; giovanni.marcenaro97@gmail.com (G.M.); marta.maccio@gmail.com (M.M.); 3 Department of Health Promotion Sciences Maternal and Infantile Care, Internal Medicine and Medical Specialities (ProMISE), University of Palermo, 90133 Palermo, Italy; ludovico.lagrutta@unipa.it; 4 Department of Radiology, AOUP Paolo Giaccone, 90127 Palermo, Italy; 5 Radiology Unit, Department of Health Promotion Sciences, IRCCS ISMETT (Mediterranean Institute for Transplantation and Advanced Specialized Therapies), 90127 Palermo, Italy; 6 Maternal and Infantile Care, Internal Medicine and Medical Specialities (ProMISE), University of Palermo, 90133 Palermo, Italy; 7 Biomedicine, Neuroscience and Advanced Diagnostics (BIND), The University Hospital Policlinico Paolo Giaccone, 90127 Palermo, Italy; 8 Department of Internal Medicine (DIMI), University of Genoa, 16126 Genoa, Italy; 9 Istituto di Elettronica e di Ingegneria dell’Informazione e delle Telecomunicazioni, Consiglio Nazionale delle Ricerche (CNR-IEIIT), 20133 Milan, Italygaia.vettori@mail.polimi.it (G.V.);; 10 Dipartimento di Elettronica, Informazione e Bioingegneria, Politecnico di Milano, 20133 Milan, Italy

**Keywords:** sarcopenia, respiratory muscles, radiomics, computed tomography, segmentation protocol, quantitative imaging, chronic lung disease

## Abstract

**Background/Objectives:** Respiratory muscle sarcopenia worsens outcomes in chronic lung disease, and quantitative Computed Tomography (CT) may provide objective biomarkers; this study aimed to develop a time-efficient segmentation protocol and identify radiomic biomarkers of respiratory muscle sarcopenia. **Methods:** This retrospective study analyzed 30 unenhanced chest CT from adult patients. The whole volume of the pectoralis major (PM), pectoralis minor (Pm), serratus anterior (SA), and fourth intercostal (4I) muscles was manually segmented. Patients were classified as sarcopenic or non-sarcopenic. Radiomics features and mean muscle density were extracted using PyRadiomics. Features associated with sarcopenia were selected using Least Absolute Shrinkage and Selection Operator (LASSO) regression and backward stepwise selection. Four sets of slices consisting of one, three, five, and seven slices were then sampled from each muscle around a fixed anatomical landmark. Deviations of each set of slices from whole-muscle metrics were evaluated using Mean Absolute Error (MAE) and Mean Absolute Percentage Error (MAPE). **Results:** Features selection identified 25 biomarkers of sarcopenia in PM, 24 in Pm, and 34 in SA. Variability-related features were significantly associated with sarcopenia (OR = 2.26; *p* = 0.012), while structural features showed an inverse association (OR = 0.18; *p* = 0.004). Mean muscle density and most radiomic features were well represented by single slice for every muscle. In the PM and Pm, eight and six radiomic features were better approximated segmenting more than one slice (*p* < 0.05). **Conclusions:** Radiomics enables quantitative assessment of sarcopenia. For SA, a simplified segmentation protocol consisting of a single slice enables approximating muscle density and radiomics of whole muscle volume. For PM and Pm, three or more slices allow a better representation of 8 and 6 radiomic features, respectively.

## 1. Introduction

Increasing efforts are devoted to the development of precise, reproducible, and time-effective methods to quantify muscular trophism in lung conditions such as Chronic Obstructive Pulmonary Disease (COPD), as low oxygen tension, metabolic changes, and the release of inflammatory mediators have been demonstrated to lead to sarcopenia up to 22% of patients [1]. In these subjects, recent studies demonstrated a link between respiratory muscles impairment and adverse outcomes, and targeted rehabilitation protocols have been suggested to arrest or reverse sarcopenia [2,3,4]. Computed Tomography (CT) may represent a potentially time-effective modality for monitoring respiratory muscles trophism, as these patients undergo multiple examinations during their disease to evaluate exacerbations, and most of the inspiratory and expiratory muscles are included in the field of view of standard examinations. In this regard, an association between the cross-sectional area of the pectoralis major and spirometric measures has been demonstrated [5], but questions remain about the appropriateness of quantifying sarcopenia by measuring a specific region of a single muscle, given that this condition can affect different muscle groups or even different parts of the same muscle in a non-uniform way. Therefore, segmentation of the entire volume of all the respiratory muscles would provide a more precise quantification of sarcopenia but appears too time-consuming to be feasible in clinical practice, and automated segmentation tools still have several limitations [6]. Additionally, a more extensive investigation of tissue microarchitecture seems relevant in pulmonary diseases, since in these conditions skeletal muscles face peculiar changes that differ from the ones characterizing sarcopenia in other diseases [7]. Radiomics quantitatively extracts data from standard-of-care medical images and can be defined as an algorithm-based quantitative analysis of image features [8]. This method has potential in providing additional details on muscle structure and has already been employed to monitor the progression of sarcopenia in conditions such as cancer, but in lung diseases its use has not been explored yet [9]. Following these considerations, the development of a time-effective and reproducible protocol enabling a comprehensive quantification of respiratory muscles trophism appears desirable. The aim of this study is twofold: (i) to develop a time-efficient protocol for extracting quantitative biomarkers of respiratory muscles sarcopenia from unenhanced chest CT; and (ii) to analyze both conventional and radiomic features derived from the full volume of respiratory muscles in order to identify the most informative biomarkers of muscle trophism.

## 2. Materials and Methods

### 2.1. Study Design and Population

Approval for this retrospective study, conducted at IRCCS Ospedale Policlinico San Martino and Policlinico Paolo Giaccone, was obtained from the competent Ethics Committee (Comitato Etico Regione Liguria; protocol code 13414, approved on 12 October 2023). Informed consent was obtained from all participants. The institutional imaging database of the Radiology Unit of the IRCCS Ospedale Policlinico San Martino was screened to identify unenhanced CTs including the thoracic region from the first thoracic to the first lumbar vertebra performed between 1 November 2023 and 12 August 2024. Patients had to be positioned with both arms raised above the head and only exams free from metallic artifacts, such as those caused by pacemakers, prostheses, or sternal wires, were included (Table 1).

All CTs were acquired using the same scanner model (dual-source, 128 × 2 slices; SOMATOM Drive, Siemens, Erlangen, Germany), kilovoltage between 120 and 130, and slice thickness of 2 mm, mAs according to patient body size, spiral pitch factor 0.98, and collimation width 0.625. The volume of the pectoralis major (PM), pectoralis minor (Pm), serratus anterior (SA), and fourth intercostal muscle (4I) was manually segmented by a radiologist with 8 years of experience until they could be distinguished from the surrounding structures using an open-source software (3D Slicer 5.8.1) and reviewed by a second radiologist with 10 years of experience. PM and Pm were segmented in the axial plane, whereas SA and 4I were segmented in the coronal plane. To exclude peripheral slices potentially affected by partial volume effects or segmentation noise, a threshold-based exclusion criterion was applied, removing slices with a number of segmented pixels lower than 20% of the maximum slice within the same muscle. In addition, slices with a z-score greater than ±3 based on the distribution of mean density values within each subject were excluded to eliminate statistical outliers. Patients’ sex, age at the time of acquisition of CT, and ethnicity were retrieved from the electronic medical charts whereas the Body Mass Index (BMI) was estimated from anthropometric data included in each exam as described in the literature [10]. Sarcopenia was defined in accordance with the quantitative muscle density thresholds established by Derstine et al. [11], who identified reference cut-off values for skeletal muscle attenuation at the level of the T1 vertebra in a healthy population. These concepts have been integrated within the conceptual framework of the European Working Group on Sarcopenia in Older People (EWGSOP) and its 2019 revision (EWGSOP2) [12]. All statistical analyses were performed using R (version 4.4.2).

### 2.2. Analysis of Muscle Density and Radiomics and Correlation with Demographics

For density characterization, statistical comparisons were conducted between sarcopenic and non-sarcopenic groups using Welch’s t-test or Mann–Whitney U-test depending on normality assumptions. Age-stratified analyses were also performed, with patients grouped into predefined age categories: 18–45 years, 46–69 years, and ≥70 years. A data-cleaning phase of radiomic features was implemented to ensure robustness and variables with zero variance or containing missing values were removed. Highly correlated features (threshold >0.9) were iteratively excluded to minimize redundancy. Each muscle dataset was processed separately to account for anatomical and structural differences. All features were normalized to ensure comparability. Feature selection followed a two-step process, beginning with LASSO regression to identify the most relevant by penalizing those with weak associations with sarcopenia status [13]. This was followed by backward stepwise selection to further refine the set of predictive variables [14]. As for LASSO selection, to determine the optimal regularization parameter (lambda), a 10-fold cross-validation approach was applied using sarcopenia classification as the target variable. A backward stepwise logistic regression based on the Akaike Information Criterion (AIC) was used to select features associated with sarcopenia. Only variables with *p* < 0.05 after backward elimination were retained. For each muscle, the selected features were standardized within groups using z-score normalization and tested for normality with the Shapiro–Wilk test. The Kruskal–Wallis test was applied to compare sarcopenic and non-sarcopenic patients, retaining only features with *p* < 0.05. Mean standardized values were then calculated for each group, and the difference (delta mean) was used to quantify changes. Significant features were finally classified into two categories—variability-related and structural-related and compared using both the Kruskal–Wallis test and a logistic regression model [15].

### 2.3. Definition of a Simplified Segmentation Protocol for Respiratory Muscles

Radiomic features (including first-order statistics, shape descriptors, and texture metrics) and mean density, this latter expressed in Hounsfield units (HU), were extracted from the whole segmented volume using an open-source tool (Python package PyRadiomics v3.0.1). Volume and segmentation mask were verified for size and spacing consistency to prevent misalignment errors. An analysis of density distribution across volume was performed for each respiratory muscle with the aim of identifying muscle regions less prone to textural heterogeneity. The Jensen-Shannon distance (JS) was calculated for each slice to compare individual slice density with the mean density of the entire muscle volume. Reproducible anatomic landmarks were identified for each muscle in regions less prone to heterogeneity based on JS analysis. After that, four sets consisting of one, three, five, and seven slices (1S, 3S, 5S, 7S) were defined, each representing a symmetrical sampling around the anatomical landmark. To account for intra-operator variability in landmark identification, the landmark position was also shifted by one and two slices forward and backward and the same sets of slices were re-sampled, resulting in five offset configurations within each group. For both radiomic features and mean density, the deviation from the reference value derived from the whole muscle volume was quantified using mean absolute error metrics, expressed as percentage error for radiomic features (MAPE) and as absolute difference in Hounsfield Units (HU) for density. To assess both landmark shifts and slice group differences, each feature was first tested for normality using the Shapiro–Wilk test. Depending on the result, either one-way ANOVA (for normally distributed data) or the Kruskal–Wallis test (for non-normal data) was applied. In the presence of significant differences, post hoc pairwise comparisons were performed using Dunn’s test with appropriate correction for multiple testing.

## 3. Results

### 3.1. Patients’ Characteristic

**Figure 1:**
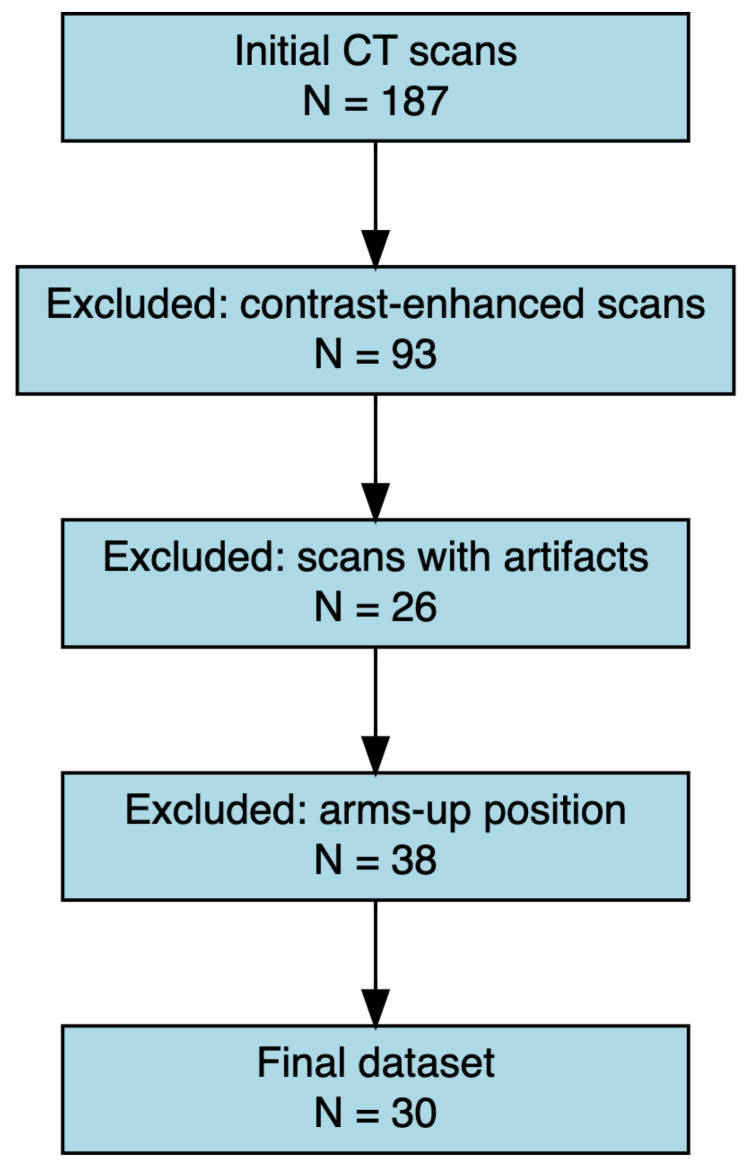
Flow diagram of the CT scan selection process. Out of 187 initial CT scans, 93 were excluded due to the presence of contrast enhancement, 26 due to imaging artifacts, and 38 due to arms-up positioning. The final dataset consisted of 30 eligible scans that met all inclusion criteria.

A total of 30 CT from 30 subjects (47% male; mean age 64.7 yr.) were included in the study (Figure 1).

Post hoc power estimation was conducted based on the observed effect size (Kendall’s W ≈ 0.3) derived from the Friedman test comparing mean density values across sampling groups (1S, 3S, 5S, 7S). The corresponding Cohen’s f value (≈0.65) indicated a large effect size, yielding a statistical power > 90% for detecting between-group differences with a significance level of 0.05. 17 patients (56.7%) were classified as sarcopenic and 13 (43.3%) as non-sarcopenic (Table 2).

**Figure 2:**
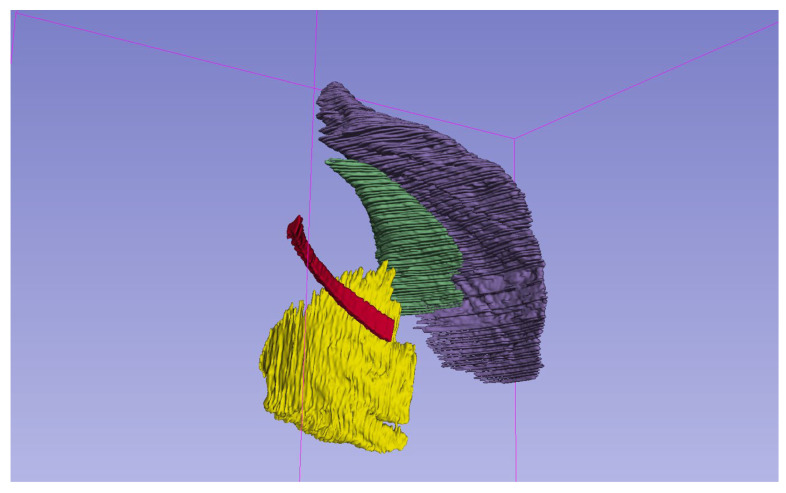
Three-dimensional reconstruction of segmented respiratory muscles from unenhanced chest CT. The pectoralis major (purple), pectoralis minor (green), serratus anterior (yellow), and IV intercostal muscle (red) are shown. Segmentations were performed using manual volumetric tracing on 3D Slicer. Each muscle is color-coded for clarity and spatially separated to highlight anatomical relationships.

After elimination of the outliers, the mean number of segmented slices was 94 for PM, 61 for Pm, 126 for SA, and 170 for 4I (Figure 2).

The time for effective whole volume segmentation of one patient-including the delineation of four muscle groups, visual quality control, and manual correction-was approximately three working days, whereas the 1S, 3S, 5S, and 7S protocols required respectively ~15 min, ~30 min, ~45 min, and ~60 min per patient.

### 3.2. Density Characterization

When comparing sarcopenic and non-sarcopenic patients, muscle density was significantly lower (*p* < 0.001) in the first group across all muscles, with the largest differences observed in PM as shown in Table 3.

A significant positive correlation was found between age and sarcopenia status (rho = 0.67, 95% CI: 0.66–0.68; *p* < 0.001). In the same way, a decline in density was observed from the 18–45 group to older age groups, particularly between 18–45 and ≥70 years, across all muscles (*p* < 0.001) (Table 4).

In contrast, no correlation between mean density and BMI was demonstrated.

### 3.3. Radiomics Features Analysis

**Figure 3:**
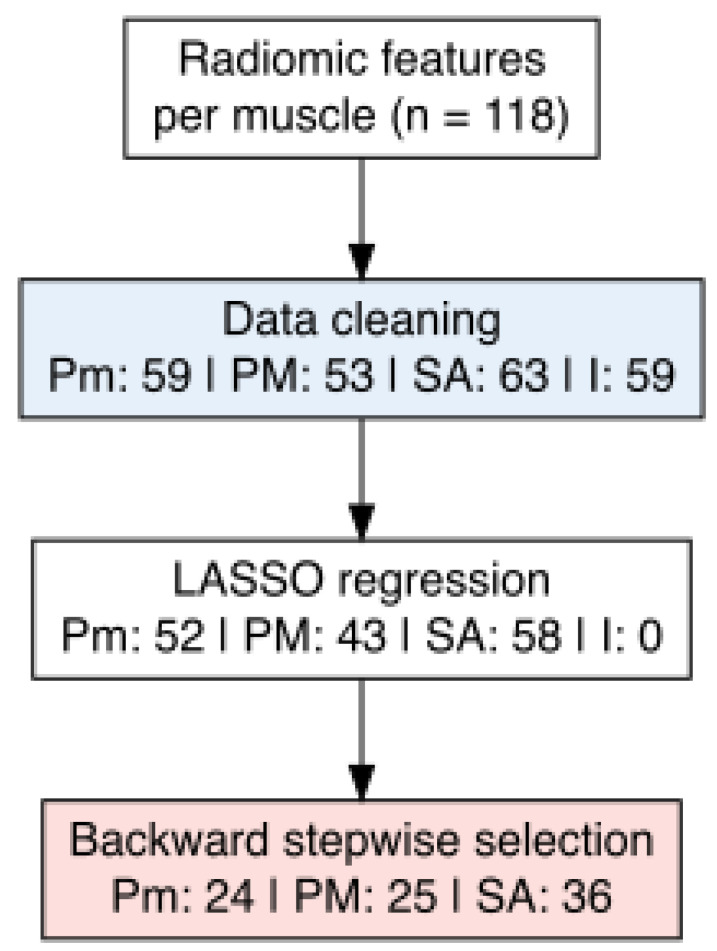
Flow diagram illustrating the feature selection workflow for the radiomic analysis of respiratory muscles. Starting from 118 features per muscle, data cleaning excluded variables with zero variance or missing values. Least Absolute Shrinkage and Selection Operator (LASSO) regression was then applied to reduce multicollinearity and identify features associated with sarcopenia. Final selection was performed using backward stepwise regression. The number of retained features at each step is reported for the pectoralis minor (Pm), pectoralis major (PM), serratus anterior (SA), and fourth intercostal muscle (4I).

After completing the feature selection pipeline, a total of 25 features for PM, 24 for Pm, and 34 for SA were retained (Figure 3, Table 5), whereas no features were ultimately selected for 4I muscle.

**Figure 4:**
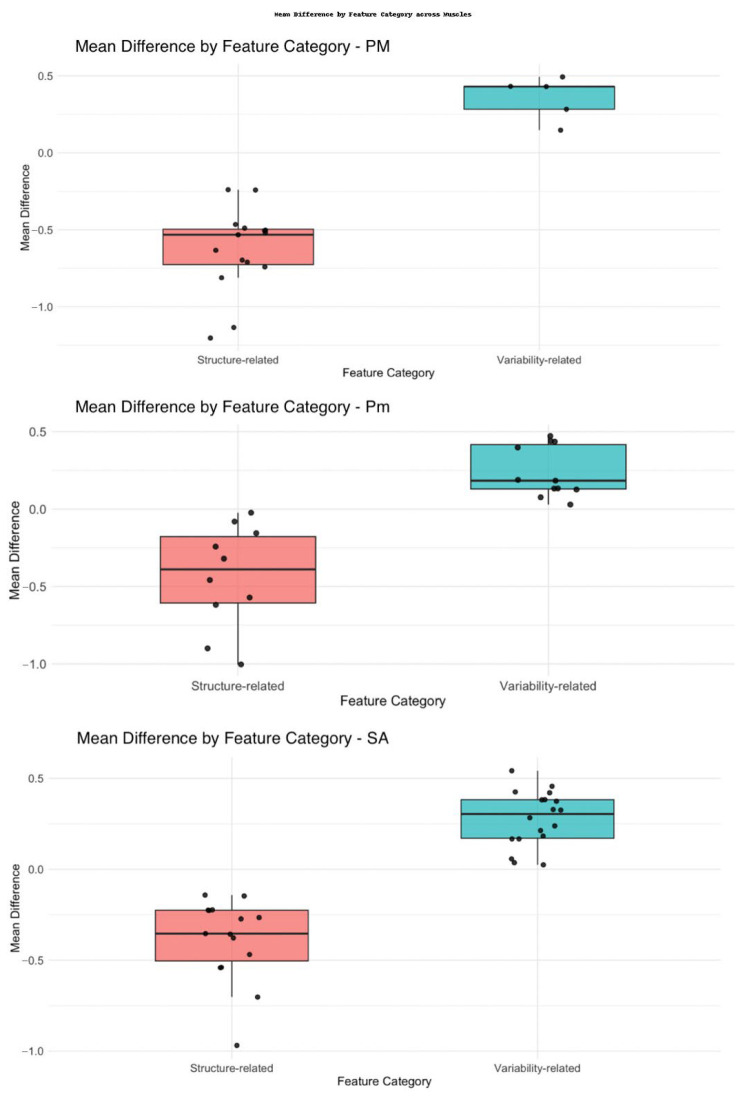
Box plots showing the mean standardized differences in radiomic features between sarcopenic and non-sarcopenic patients for three respiratory muscles: pectoralis major (PM), pectoralis minor (Pm), and serratus anterior (SA). Features are categorized as structure-related (red) and variability-related (green). Each dot represents an individual radiomic feature. PM = pectoralis major; Pm = pectoralis minor; SA = serratus anterior.

Both shape descriptors, such as elongation and maximum diameter, and first-order statistics, including median and robust mean absolute deviation, showed substantial reductions in sarcopenic patients, reflecting volumetric and density alterations. A distinct pattern of radiomic features variation between sarcopenic and non-sarcopenic patients was observed for the PM, Pm, and SA, with a consistent trend of increasing of variability-related features and decreasing of structure-related characteristics in sarcopenic individuals (Figure 4).

In addition, the logistic regression model revealed that higher values in the variability-related cluster were significantly associated with increased odds of sarcopenia (OR = 2.26, 95% CI: 1.39–4.06, *p* = 0.012), while higher values in the structure-related cluster were inversely associated with sarcopenia (OR = 0.18, 95% CI: 0.05–0.56, *p* = 0.004).

### 3.4. Identification of Anatomic Landmarks and Definition of Segmentation Protocol

Slices in the central regions of each muscle consistently exhibited lower JS distances compared to peripheral ones. Moreover, slices with high JS distances were often surrounded by adjacent slices with similarly elevated divergence, suggesting spatial clustering of local variability. Based on this observation, the sternoclavicular joint was chosen as landmark for the segmentation of PM and Pm in the axial plane, whereas the first costovertebral joint was identified as landmark for segmenting SA and 4I in the coronal plane. From these landmarks the four different volume samples were extracted from each muscle, with five slices interposed in between to avoid high-variability clusters.

### 3.5. Comparison of Density and Radiomics in Small Slice Sets and in the Entire Muscle Volume

**Figure 5:**
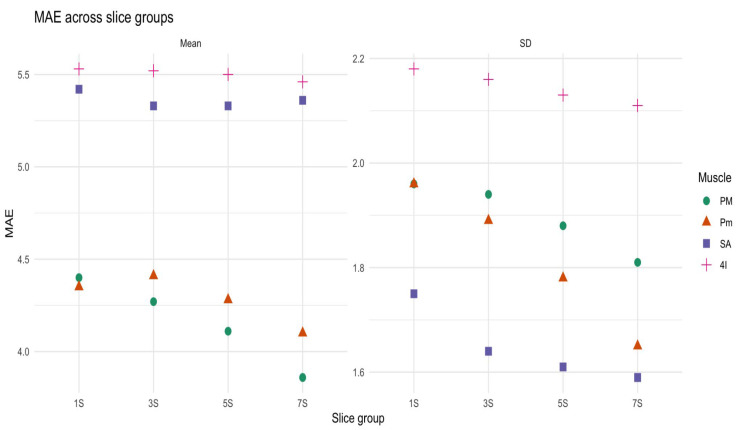
Mean absolute error (MAE) of mean density and standard deviation (SD) across slice groups (1S, 3S, 5S, 7S) for four respiratory muscles: pectoralis major (PM, green circles), pectoralis minor (Pm, orange triangles), serratus anterior (SA, blue squares), and fourth intercostal muscle (4I, pink crosses).

Shifts in the position of the anatomical landmark did not result in significant differences in mean density (*p* = 0.47) and SD (*p* = 0.1) across configuration. The comparison of MAE across the four sets of slices showed modest differences in all muscles and metrics. with Kruskal–Wallis tests not revealing significant differences between slice groups for mean density and SD (e.g., PM: Mean *p* = 0.45, SD *p* = 0.08). Notably, the MAE for the mean density remained below 6 HU across all muscles and slice groups (Figure 5).

**Figure 6:**
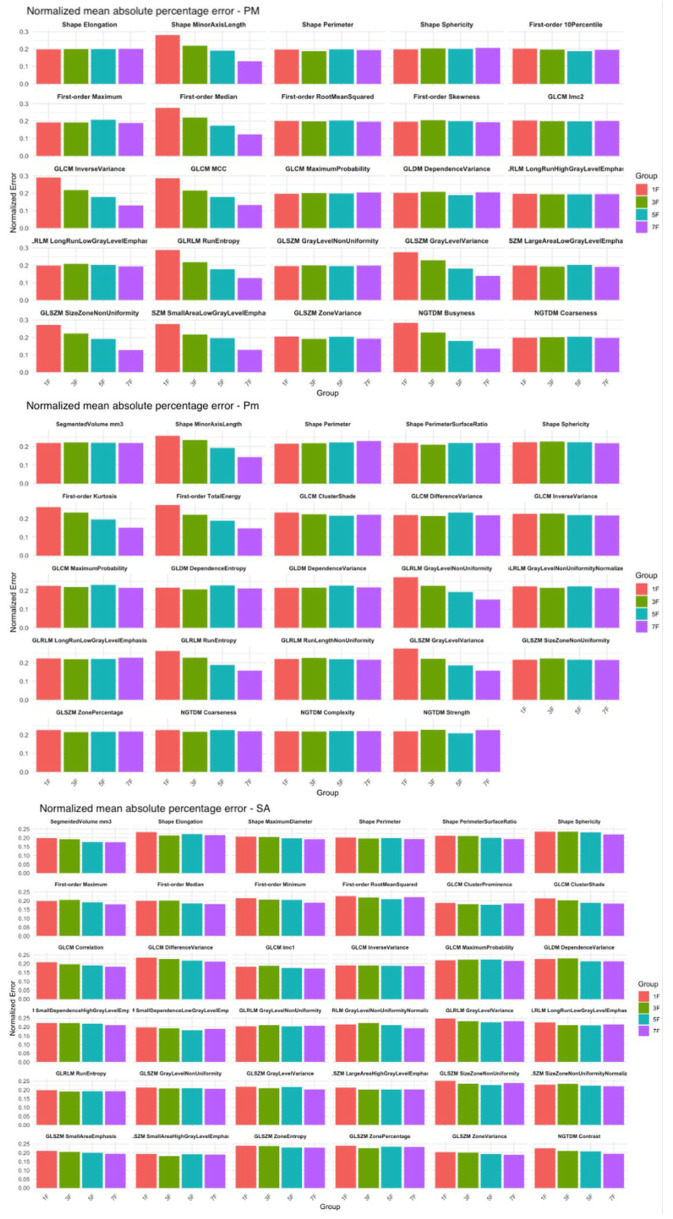
Normalized mean absolute percentage error (MAPE) of radiomic features for the pectoralis major (PM), pectoralis minor (Pm), and serratus anterior (SA) muscles, calculated across four slice sampling groups: 1S (red), 3S (green), 5S (blue), and 7S (purple). The analyzed features include first-order statistics (e.g., Maximum, Skewness, Total Energy), shape descriptors (e.g., Elongation, Sphericity, Perimeter), and texture features derived from the gray level co-occurrence matrix (GLCM), gray level run length matrix (GLRLM), gray level size zone matrix (GLSZM), gray level dependence matrix (GLDM), and neighboring gray tone difference matrix (NGTDM).

In the PM and Pm, a statistically significant difference (*p* < 0.05) between the 1S group and all other groups was observed for 8 and 6 features respectively (Figure 6). For all the other radiomics features, increasing the number of sampled slices did not result in a significant reduction in the MAPE. Conversely, for the SA, the radiomics features remained stable across all sampled groups, with no statistically significant differences observed between groups (Figure 6).

A decrease in SD was also observed across groups, particularly between 1S and 3S in PM and Pm. Post hoc tests confirmed statistically significant differences between the 1S group and the 3S, 5S, and 7S groups for 16 features in PM and 13 in Pm.

## 4. Discussion

This study explored both muscle density and radiomic features to characterize sarcopenia in patients undergoing unenhanced chest CT and to design a reproducible, time-efficient segmentation protocol for respiratory muscles. We found that sarcopenia was associated with both a reduction in average muscle density and significant differences in radiomic features in three of the respiratory muscles analyzed, whereas radiomics analysis of 4I did not reveal any features correlated with sarcopenia. As for the density reduction, this trend confirmed previous evidence on the potential of CT in detecting fat infiltration and muscle degradation in this condition [15,16]. Radiomic analysis revealed that sarcopenia was linked to an increase in variability-related features (e.g., texture heterogeneity) and a decrease in structure-related features (e.g., shape and first-order density metrics) in the PM, Pm, and SA. Interestingly, no radiomic features from 4I were retained after feature selection, suggesting that this muscle may be less informative for detecting sarcopenia-related changes. A possible explanation lies in the structural characteristics of the intercostal muscles and the difficulties in their segmentation. In fact, these structures appear in CT scans as a few muscle fibers located between the lung parenchyma, ribs, and adipose tissue and even minor errors in segmentation may significantly alter the results of muscle analysis, thus impairing the potential of radiomics in disclosing differences between sarcopenic and non-sarcopenic patients. Our findings align with earlier research that explored the potential of radiomics in detecting changes in pectoralis muscle trophism and investigated the associations between sarcopenia-related features and clinical outcomes in chronic conditions such as cancer and diabetes [17,18,19]. On the other hand, less information is available regarding the potential role of radiomic biomarkers extracted from SA in characterizing respiratory muscle atrophy and failure. However, in the present study, radiomics analysis of SA found 34 features that were altered in sarcopenic patients—more than those observed in PM and Pm. Based on these findings and on the relevant role that this muscle plays as an accessory respiratory muscle, it may be advisable to include SA analysis in future studies on sarcopenia in chronic lung diseases, as it may provide additional outcomes measures not obtainable from PM and Pm evaluation.

Based on a systematic evaluation of the mean density across different parts of muscle volume, we identified the sternoclavicular joint (for PM and Pm) and the first costovertebral joint (for SA and 4I) as optimal anatomical landmarks for respiratory muscles segmentation. Our data show that for SA and 4I muscles, a single coronal slice centered on the landmark is sufficient to approximate the radiomic and density characteristic of the whole muscle volume. In contrast, segmenting only one slice of PM and Pm introduces a significant increase in MAPE of eight and six radiomics features, respectively, whereas extracting three slices while skipping five slices between each proved effective results in mitigating the impact of local heterogeneity. This approach also resulted in a substantial time reduction: while full-volume manual segmentation required three working days per patient, the simplified protocol allowed segmentation of four respiratory muscles in less than one hour per patient. With the advancement of precision medicine and the increasing need of standardized, reproducible, and sensitive biomarkers able to detect minor changes after therapies, our results may provide an effective guide for future works addressing the investigation of respiratory muscles trophism through CT. Furthermore, we demonstrated that shifting the landmark position by one or two slices forward or backward did not significantly alter the radiomic measurements, especially in multi-slice groups (3S, 5S, 7S). This robustness to small positional shifts confirms the reproducibility of our method and supports its applicability in clinical and research settings, where slight variations in landmark placement are inevitable. These findings address a common limitation in radiomic analysis, operator dependence, and offer a feasible compromise between complete manual segmentation and automated approaches that present several limitations and are not fully implemented in clinical practice [6,20].

As with any exploratory study, some methodological and contextual limitations emerged. First, the sample size, although consistent with pilot radiomic studies, may limit generalizability. However, we adopted a rigorous imaging protocol, excluded artifacts, standardized CT acquisition parameters, and applied robust statistical and machine learning techniques (LASSO and backward selection) to enhance internal validity. In addition, although we included four respiratory muscles, other accessory or core muscles were not analyzed. Finally, more data is needed to investigate the potential overlap of information provided through standard and radiomics analysis of each respiratory muscle.

## 5. Conclusions

In conclusion, our study provides a comprehensive assessment of sarcopenia-related changes in respiratory muscles using CT-based density and radiomic analysis. We identified a set of reproducible and discriminative biomarkers and proposed a practical segmentation protocol adaptable to clinical workflows. This protocol may help standardize radiomic evaluation of respiratory muscles across centers and facilitate early identification of sarcopenia in high-risk pulmonary patients. These findings support the integration of radiomic biomarkers into routine chest CT interpretation, offering a feasible pathway to identify sarcopenia early and optimize patient-specific rehabilitation strategies.

## Figures and Tables

**Table 1:** Inclusion criteria.

Inclusion Criteria	Exclusion Criteria
Unenhanced CT scans including thoracic region from T1 to L1	Presence of metallic artifacts (e.g., pacemakers, prostheses, sternal wires)
Patient positioned with both arms raised above the head	Age < 18 years
CT acquired with the same scanner model	Poor image quality
Kilovoltage set between 120 and 130 kV	
Slice thickness of 2 mm	

**Table 2:** Baseline characteristics of the study population, stratified by sarcopenia status. Data are presented as mean ± standard deviation (SD) for continuous variables and as number (percentage) for categorical variables. BMI = Body Mass Index; SD = Standard Deviation.

Variable	Total (*n* = 30)	Sarcopenic (*n* = 17)	Non-Sarcopenic (*n* = 13)
Age (years)	64.7 ± 17.2	70.4 ± 12.5	56.1 ± 20.0
BMI (kg/m^2^)	23.1 ± 3.3	22.0 ± 2.9	24.9 ± 3.2
Females (*n*)	16 (53%)	11 (65%)	5 (38%)
Males (*n*)	14 (47%)	6 (35%)	8 (62%)
Caucasian	28 (93.3%)	17 (100%)	11 (85%)

**Table 3:** Muscle density values (mean ± standard deviation, in Hounsfield Units) for the total population and stratified by sarcopenia status. SA = serratus anterior; PM = pectoralis major; 4I = fourth intercostal muscle; Pm = pectoralis minor.

Muscle	Total Density (HU)	Non-Sarcopenic Density (HU)	Sarcopenic Density (HU)
PM	25.5 ± 19.9	36.9 ± 14.1	15.2 ± 18.8
Pm	27.6 ± 15.4	32.8 ± 15.9	23.3 ± 13.6
SA	15.0 ± 21.5	23.9 ± 16.6	3.1 ± 21.6
4I	−27.8 ± 26.3	−18.4 ± 25.3	−38.8 ± 23.0

**Table 4:** Muscle density values (mean ± standard deviation, in Hounsfield Units) across different age groups. SA = serratus anterior; PM = pectoralis major; 4I = fourth intercostal muscle; Pm = pectoralis minor.

Muscle	Density 18–45 Years (HU)	Density 46–69 Years (HU)	Density ≥ 70 Years (HU)
PM	44.3 ± 9.2	17.1 ± 23.9	14.2 ± 10.9
Pm	41.3 ± 11.8	22.9 ± 12.3	20.7 ± 9.1
SA	34.2 ± 7.7	9.1 ± 13.5	8.7 ± 18.7
4I	7.1 ± 11.9	−37.9 ± 12.6	−35.4 ± 20

**Table 5:** List of variability-related and density-related radiomic features selected for each muscle. Variability-related features include texture metrics derived from the gray level co-occurrence matrix (GLCM), gray level dependence matrix (GLDM), gray level run length matrix (GLRLM), gray level size zone matrix (GLSZM), and neighborhood gray tone difference matrix (NGTDM). Density-related features include first-order statistics and segmented muscle volume (mm^3^). LRLM indicates long run length matrix.

Muscle	Variability-Related Features	Density-Related Features
Pectoralis Major (PM)	GLCM Inverse Variance, GLCM MCC, GLCM Maximum Probability, GLDM Dependence Variance, GLRLM Run Entropy, GLSZM Gray Level Non-Uniformity, GLSZM Gray Level Variance, GLSZM Zone Variance, GLSZM Size Zone Non-Uniformity, NGTDM Busyness, NGTDM Coarseness, RLM Long Run Low Gray Level Emphasis, GLCM Imc2	First-order Maximum, First-order Median, First-order Root Mean Squared, First-order Skewness, First-order 10Percentile
Pectoralis Minor (Pm)	GLCM Cluster Shade, GLCM Difference Variance, GLCM Inverse Variance, GLCM Maximum Probability, GLDM Dependence Entropy, GLDM Dependence Variance, GLRLM Gray Level Non-Uniformity, GLRLM Run Entropy, GLRLM Run Length Non-Uniformity, GLSZM Gray Level Variance, GLSZM Size Zone Non Uniformity, GLSZM Zone Percentage, NGTDM Coarseness, NGTDM Complexity, NGTDM Strength, LRLM Gray Level Non Uniformity Normalized	First-order Kurtosis, First-order Total Energy, Segmented Volume mm^3^
Serratus Anterior (SA)	GLCM Cluster Prominence, GLCM Cluster Shade, GLCM Correlation, GLCM Difference Variance, GLCM Inverse Variance, GLCM Maximum Probability, GLDM Dependence Variance, GLRLM Gray Level Non-Uniformity, GLSZM Gray Level Variance, GLSZM Zone Entropy, GLSZM Zone Variance, GLSZM Size Zone Non-Uniformity, GLSZM Zone Percentage, NGTDM Contrast, NGTDM Coarseness	First-order Maximum, First-order Median, First-order Minimum, First-order Root Mean Squared, Segmented Volume mm^3^

## Data Availability

The datasets generated and analyzed during the current study are available from the corresponding author on reasonable request.

## Abbreviations

The following abbreviations are used in this manuscript:

BMI	Body Mass Index
COPD	Chronic Obstructive Pulmonary Disease
CT	Computed Tomography
HU	Hounsfield Units
LASSO	Least Absolute Shrinkage and Selection Operator
MAE	Mean Absolute Error
MAPE	Mean Absolute Percentage Error
OR	Odds Ratio
Pm	Pectoralis Minor
PM	Pectoralis Major
SA	Serratus Anterior
SD	Standard Deviation
4I	Fourth intercostal muscle
